# Reform in Medical Colleges

**Published:** 1867-06

**Authors:** 


REFORM IN MEDICAL COLLEGES.

Dr. Sayre, of New York, offered the following:

*Resolved*, That this Association, most heartly approving the whole action of the Convention of Medical Colleges, urge its practical adoption on all the Medical Colleges in the country.

Dr. Post, of New York, wished to offer some change in the course of instruction proposed.

Dr. Davis cut the gentleman off by saying that the College to which he was attached had been invited to be present and take part in the proceedings of that Convention, but had not seen fit to do so. He could at the next meeting of that Convention offer any amendment he chose, but this Association has no power to act in the matter.

Dr. C. A. Lee, of Buffalo, though not present at the Medical

College Convention, approved of all that was done. He had for many years, as a teacher in a Medical College, contemplated just such changes as had been recommended.

The resolution was then adopted.

